# Developmental Changes in Sleep Spindle Characteristics and Sigma Power across Early Childhood

**DOI:** 10.1155/2016/3670951

**Published:** 2016-03-27

**Authors:** Ian J. McClain, Caroline Lustenberger, Peter Achermann, Jonathan M. Lassonde, Salome Kurth, Monique K. LeBourgeois

**Affiliations:** ^1^Department of Integrative Physiology, University of Colorado Boulder, Boulder, CO 80309, USA; ^2^Child Development Center, University Children's Hospital Zurich, 8032 Zurich, Switzerland; ^3^Department of Psychiatry, University of North Carolina at Chapel Hill, Chapel Hill, NC 27599, USA; ^4^Institute of Pharmacology and Toxicology, University of Zurich, 8057 Zurich, Switzerland

## Abstract

Sleep spindles, a prominent feature of the non-rapid eye movement (NREM) sleep electroencephalogram (EEG), are linked to cognitive abilities. Early childhood is a time of rapid cognitive and neurophysiological maturation; however, little is known about developmental changes in sleep spindles. In this study, we longitudinally examined trajectories of multiple sleep spindle characteristics (i.e., spindle duration, frequency, integrated spindle amplitude, and density) and power in the sigma frequency range (10–16 Hz) across ages 2, 3, and 5 years (*n* = 8; 3 males). At each time point, nocturnal sleep EEG was recorded in-home after 13-h of prior wakefulness. Spindle duration, integrated spindle amplitude, and sigma power increased with age across all EEG derivations (C3A2, C4A1, O2A1, and O1A2; all *p*s < 0.05). We also found a developmental decrease in mean spindle frequency (*p* < 0.05) but no change in spindle density with increasing age. Thus, sleep spindles increased in duration and amplitude but decreased in frequency across early childhood. Our data characterize early developmental changes in sleep spindles, which may advance understanding of thalamocortical brain connectivity and associated lifelong disease processes. These findings also provide unique insights into spindle ontogenesis in early childhood and may help identify electrophysiological features related to healthy and aberrant brain maturation.

## 1. Introduction

Sleep spindles are a prominent feature of the sleep encephalogram (EEG) during non-rapid eye movement (NREM) sleep [1]. In adults, spindles are commonly characterized as rhythmic bursts of activity in the 10–16 Hz frequency range lasting at least 0.3 s in duration [1–3]. In the context of visually scored sleep stages, spindles are most prevalent in NREM stage 2 sleep [1, 4, 5]. Sigma activity and sleep spindle characteristics can be examined by means of spectral analysis, visual identification, and/or automated detection methods. Of note, during NREM stages 3 and 4 sleep, discrete spindles are often masked by pronounced slow waves, thus making visual detection during slow wave sleep challenging and supporting the need for automatic detection approaches [6, 7]. This is especially true in childhood, a period during which the amplitude of slow waves is highest across the lifespan [8].

Sleep spindles are consistently linked to cognitive abilities, including working memory and problem solving [9], learning efficiency [10, 11], and memory consolidation [12, 13]. Furthermore, sigma activity is associated with fluid intelligence [14] and processing speed [15]. Data from a growing number of studies indicate sleep spindle abnormalities in individuals with mental retardation [16], autism spectrum disorder [17], neurodegenerative disease [18], sleep disorders [19], and mental illness [20–22]. For example, patients with schizophrenia have reduced spindle density, amplitude, and duration compared to healthy controls [23, 24]. In the case of autism spectrum disorder, patients have reduced sleep spindles compared to healthy individuals [17]. As such, Limoges and colleagues [17] hypothesized that abnormal spindle characteristics may be related to alterations in cortical column morphology and cell distribution, which subsequently alters cortical connectivity and leads to dysregulated thalamocortical feedback. Although the mechanisms underlying these disease processes remain to be elucidated, sleep spindles provide insights into the potential role of disrupted neuronal circuits in the thalamus and cerebral cortex.

In addition to disease, sleep spindles may be useful biomarkers for neurodevelopment. The infant sleep EEG is characterized by the presence of sleep spindles during quiet sleep (analogous to NREM sleep), which appear as early as 4–9 weeks of age [25]. According to one cross-sectional study, spindle density (number of spindles/time) exhibits an inverted U-shaped time course during infancy and the toddler years (i.e., an increase from 9 weeks until ~6 months of age followed by a decrease, with a minimum between ages 20–28 months), followed by an increase across early childhood [26]. This study, however, did not account for the high interindividual variability in sleep spindle measures [6, 26] and restricted spindle identification to short episodes of NREM sleep. Therefore, although published findings suggest age-related changes in functional brain connectivity within the thalamo-cortical network [27], longitudinal studies that account for individual differences in spindle characteristics across early childhood remain scarce.

Sleep spindles are associated with several disease processes and may aid clinicians in evaluating the integrity of thalamocortical circuits. As spindle characteristics may also serve as markers of cortical and subcortical brain growth, describing their developmental trajectories in healthy children starting in early life is a fundamental step to expand knowledge of sleep physiology and the maturing brain. In this study, we utilized a well-controlled sleep protocol at 3 longitudinal time points between the toddler and kindergarten years. At each age, we quantified several spindle characteristics (i.e., frequency, density, duration, and integrated spindle amplitude (ISA)) using an age-adapted state-of-the-art automated detection algorithm and sigma power with spectral analysis during NREM sleep. With these data, we examined developmental changes in each measure during early childhood, a sensitive window in the maturation of cognitive abilities, sleep, and the brain.

## 2. Methods

### 2.1. Subjects

Eight healthy children (3 males; 6 Caucasians and 2 mixed race) were longitudinally assessed at three time points: 2 years (2 Y; 2.8 ± 0.2 y), 3 years (3 Y; 3.8 ± 0.2 y), and 5 years (5 Y; 5.9 ± 0.2 y). Children were of healthy weight for age and height (i.e., 5th–85th percentile BMI) and were from middle to upper-middle class families based on parental education, occupation, and family income. These demographic factors were stable across time. Subjects were excluded from the study on the basis of prior travel, medication use, sleep problems, developmental or cognitive disabilities, head injury, low birth weight, or the presence of neurologic or other chronic disorders (exclusion criteria are detailed in our previous publication [27]). For the duration of the study, subjects were not allowed to consume caffeine or medications affecting sleep or arousal. At 2 Y all children napped regularly, at 3 Y most children were still napping, and at 5 Y all subjects had stopped napping (*n* = 7). Parents were compensated with cash, and children were given small nonmonetary gifts throughout the course of the study. Experimental procedures were explained to parents before they provided written informed consent as approved by the Institutional Review Board at Brown University. Study procedures were performed according to the Declaration of Helsinki.

### 2.2. Sleep EEG Assessments

Following a 5-day sleep-wakefulness stabilization schedule, all-night sleep EEG was recorded after 13-h of prior wakefulness in subject's homes. Children slept in their typical sleep environment during both the lead-in stabilization period and during the overnight sleep EEG recording. The stabilization schedule of younger children (2 Y and 3 Y) required a 12.5-h sleep opportunity, including a 45-min daytime nap opportunity; older children (5 Y) were allowed a nocturnal 12-h sleep opportunity. Adherence to the study protocol was verified by wrist actigraphy (AW 64, MiniMitter, Bend, OR, USA), sleep diaries, and daily communication with parents.

Sleep EEG (derivations C3A2, C4A1, O1A2, and O2A1; International 10–20 System [28]), electromyogram (EMG), and electrooculogram (EOG) were recorded (128 Hz sampling rate at 2 Y; 256 Hz sampling rate at 3 Y and 5 Y) during one night of sleep at each time point (2 Y, 3 Y, and 5 Y) after 13-h of prior wakefulness (Vitaport 3 EEG recorder; Temec Instruments). Sleep stages were scored according to standard criteria (30-s epochs, C3A2 [29]). To examine sigma power, spectral analysis was performed for each derivation (Fast Fourier Transform; Tukey Window *r* = 0.5, mean of ten 4-s pages overlapping by 1-s; 0.25 Hz frequency resolution; VitaScore, Temec Instruments, Kekrade, Netherlands); however, only frequencies between 10 and 16 Hz were used for this analysis, a commonly defined range for sigma activity [30, 31].

### 2.3. Spindle Detection

**Figure 1:**
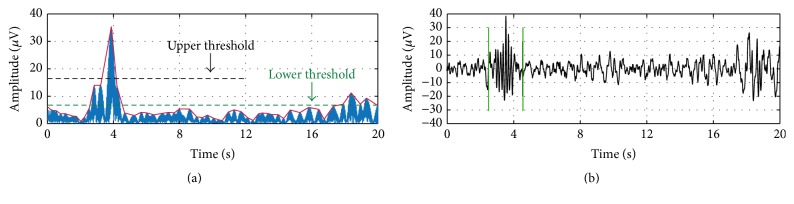
Sample spindle detection envelope (with upper and lower thresholds labeled) and raw signal. (a) Resampled (128 Hz), band pass-filtered (10–16 Hz), and rectified EEG signal. (b) Raw EEG signal with detected sleep spindle in non-rapid eye movement sleep.

For sleep spindle detection, we used an established algorithm [23] and further optimized settings as described in [2]. To summarize, the EEG signal was resampled to 128 Hz (for recordings at 3 Y and 5 Y), band pass-filtered between 10 and 16 Hz (Chebychev filter of order 6 with passband corner frequencies of 11 Hz and 15 Hz and stopband corner frequencies of 10 Hz and 16 Hz), and rectified. The algorithm then detects sleep spindles based on upper threshold (UT) and lower threshold (LT) amplitude criteria generated from the filtered signal (Figure 1). The UT was selected as 6 and the LT as 2 times the mean signal amplitude because these values provided the greatest agreement when automatically detected spindles were plotted and visually confirmed across random subjects and night segments. Additionally, Warby and colleagues found optimal detection performance at 6 times the mean after the UT value was modified from original publication [2].

Across all derivations and for each identified spindle, we calculated mean spindle frequency (Hz), duration (s), integrated spindle amplitude (ISA; *µ*V; calculated as the sum of amplitudes of the band-passed, rectified signal of each spindle as in [11]), and spindle density (number of spindles/min) for the maximum common duration of NREM sleep across subjects (688 min). Because several variables showed nonnormal distributions, we performed both parametric and nonparametric tests; however, the results did not differ between approaches. Thus, we present only parametric statistics. We examined developmental changes in time in bed (lights-off to lights-on), total sleep time (stage 2 sleep onset to sleep offset), sleep stage variables (min, %), and spindle characteristics with one-way repeated measures ANOVA, followed by* post hoc* paired *t*-tests with a Bonferroni correction (*p* < 0.016) for multiple comparisons when the ANOVA was significant. We also utilized one-way repeated measures ANOVA to assess developmental changes in spectral EEG sigma power (10–16 Hz; 0.25 Hz bins; *p* values were adjusted according to the linear step-up procedure to control for multiple comparisons [32]); paired *t*-tests with a Bonferroni correction (*p* < 0.016) were then used to determine sigma power differences between ages following a significant ANOVA.

## 3. Results

As shown in Table 1, almost all sleep variables were temporally stable across early childhood when assessing the entire nighttime sleep episode. We observed an increase in % of stage 1 sleep (*F*(2,14) = 3.9, *p* = 0.045) between 2 Y and 3 Y, as well as a decrease in REM min (*F*(2,14) = 5.3, *p* = 0.020) and REM% (*F*(2,14) = 4.0, *p* = 0.042) that emerged by the age of 5 Y (i.e., decreased between 2 Y and 5 Y).

### 3.1. Developmental Changes in Sleep Spindle Characteristics

**Figure 2:**
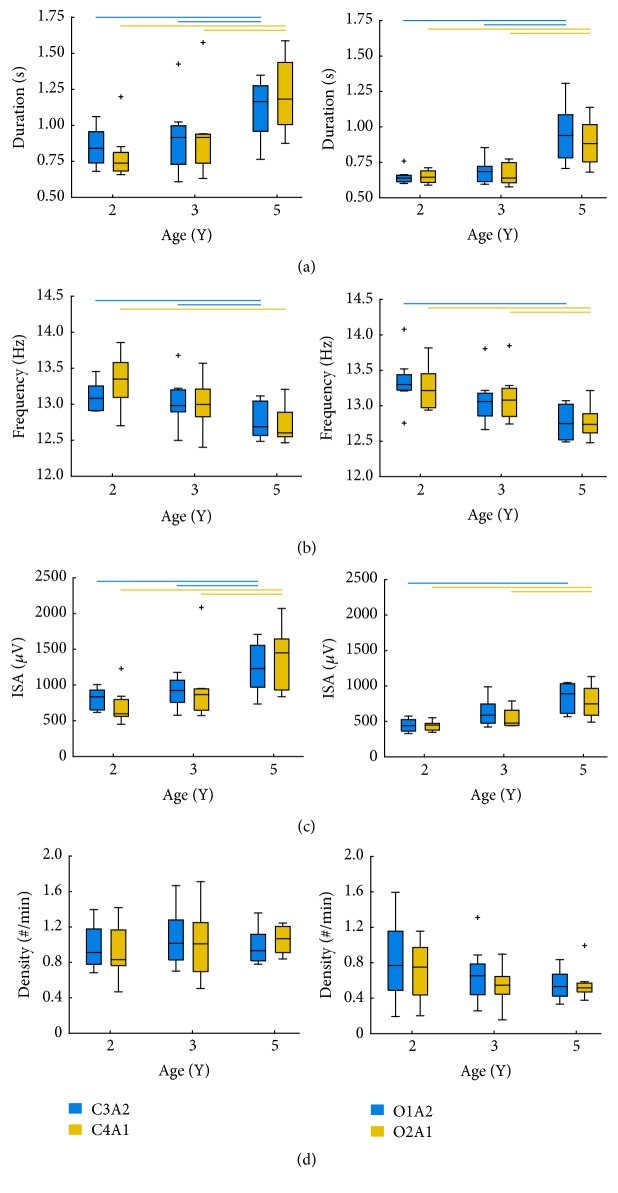
Boxplots of sleep spindle duration (a), frequency (b), integrated spindle amplitude (ISA; (c)), and density (d) at ages 2, 3, and 5 years for each EEG derivation (C3A2, C4A1, O1A2, and O2A1). Upper and lower borders of boxplots represent upper and lower quartiles, tails represent minimum and maximum scores, the vertical midline represents the median, and outliers are indicated by +. Lines at the top of each plot denote significant* post hoc* paired *t*-test comparisons (Bonferroni correction; *p* < 0.016).

We found a developmental change in the duration of sleep spindles in both central and occipital derivations (Table 2; Figure 2).* Post hoc* tests showed that spindle duration in all regions was similar between ages 2 Y and 3 Y, with an increase in spindle duration appearing by the age of 5 Y (i.e., significant differences between 2 Y versus 5 Y and 3 Y versus 5 Y; Figure 2(a)). Inspection of spindle duration across all derivations and ages showed that the shortest mean spindle duration occurred at 2 Y (0.64 ± 0.05 s; O2A1) and the longest at 5 Y (1.21 ± 0.26 s; C4A1). Across all regions, spindle duration increased on average by ~40% between ages 2 Y and 5 Y.

As shown in Table 2 and Figure 2(b), spindle frequency decreased with increasing age; however,* post hoc* analyses showed that, across all derivations, spindle frequency was similar between 2 Y and 3 Y. Similar to duration, the frequency of spindles did not show a developmental decline until the age of 5 Y. The range in spindle frequency was 0.63 Hz (12.72 Hz in C4A1 at 5 Y; 13.35 Hz in O1A2 at 2 Y). Across all regions and time points, spindle frequency decreased by ~4%.

With regard to ISA, we observed a change in all derivations with advancing age (Figure 2(c); Table 2). Specifically,* post hoc* analyses showed that ISA did not differ between ages 2 Y and 3 Y; however, a developmental increase emerged across all regions by the age of 5 Y. Overall, ISA ranged from 436.4 *µ*V (O1A2 at 2 Y) to 1357.6 *µ*V (C4A1 at 5 Y). Average ISA across all derivations increased by ~78% between ages 2 Y and 5 Y.

As illustrated in Figure 2(d) and Table 2, spindle density ranged from 0.54 (O1A2 at 3 Y) to 1.06 spindles/min (C4A1 at 5 Y); however, we found no developmental changes in sleep spindle density in any derivation (all *p*s > 0.05).

### 3.2. Maturation of Sigma Power

**Figure 3:**
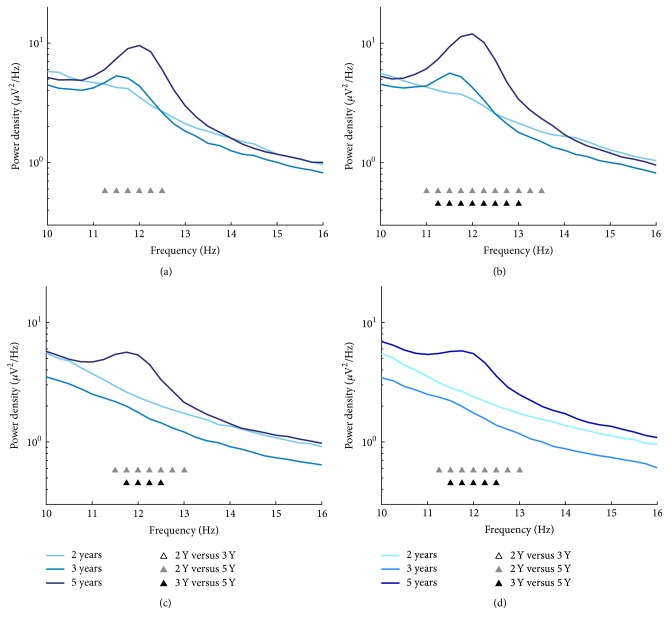
Mean spectral EEG power density spectra for the sigma range (10–16 Hz; 0.25 Hz bins) at ages 2, 3, and 5 years at C3A2 (a), C4A1 (b), O1A2 (c), and O2A1 (d). Significant* post hoc* comparisons between ages are denoted for each frequency bin.

Analysis of sigma power was performed across a wide frequency window (10–16 Hz; 0.25 Hz bins) and indicated developmental changes within 11.25–12.50 Hz in derivation C3A2 (Figure 3(a); *F* = 6.7–9.4, *η* ^2^ = 0.20–0.26, and all *p*s = 0.038) and within 11.00–13.50 Hz in C4A1 (Figure 3(b); *F* = 5.5–16.3, *η* ^2^ = 0.21–0.37, and* p* = 0.004–0.04). Sigma power also differed across age in occipital EEG regions within 11.50–13.00 Hz in O1A2 (Figure 3(c); *F* = 7.7–13.3, *η* ^2^ = 0.38–0.53, and* p* = 0.01–0.02) and within 11.25–13.00 Hz in O2A1 (Figure 3(d); *F* = 6.0–11.3, *η* ^2^ = 0.25–0.44, and* p* = 0.01–0.04). Similar to other sleep spindle characteristics,* post hoc* analyses showed that the developmental changes in sigma power emerged at the age of 5 Y (i.e., between 2 Y and 5 Y and 3 Y and 5 Y).

## 4. Discussion

This well-controlled study characterized the development of sleep spindle features and sigma power between ages 2 and 5 years. With a longitudinal approach and an age-adapted, automated spindle detection algorithm, we provide a comprehensive and objective analysis of sleep spindle trajectories across the early childhood years. Results indicate temporal stability in all spindle characteristics between ages 2 and 3 years, with a developmental shift in most features emerging by the age of 5 years (i.e., increase in spindle duration and ISA, decrease in spindle frequency, and increase in sigma power in the slower frequencies). Overall, these fundamental findings indicate maturational changes in multiple dimensions of sleep spindles, thus providing novel insights into understanding the formation and fine-tuning of neural networks (i.e., thalamocortical) across early childhood.

### 4.1. Sleep Spindle Characteristics

Multidimensional characteristics of sleep spindles (i.e., duration, frequency, amplitude, and density) can be measured, and their distinction is important: different characteristics may reflect distinct structural and functional aspects of the brain [1, 33]. For example, sleep spindle density likely mirrors the pace-making activity of the thalamic reticular nucleus, whereas duration and amplitude are further influenced by thalamocortical and corticothalamic projections [33, 34]. Additionally, ISA (which reflects the “intensity” of a sleep spindle) is a sensitive, cumulative measure of sleep spindle activity. Although multicollinearity between ISA and spindle duration and amplitude is inherent based upon ISA computation, our data indicate little overlap between other measures. For example, at the age of 2 Y (C3A2), correlations between ISA and density (*r* = −0.09, *p* = 0.85), duration and density (*r* = −0.39, *p* = 0.33), and maximum amplitude and density (*r* = −0.10; *p* = 0.81) were not significant. Although the diverse functional roles of sleep spindle characteristics are not fully understood in childhood, independent sleep spindle measures may provide more detailed information about cortical and subcortical brain development.

Evidence from* in vivo* and computational models suggests that corticothalamic feedback plays an active role in controlling the duration of sleep spindles [33]. We observed a developmental increase in sleep spindle duration that ranged from ~31% (C3A2) to ~53% (C4A1). Thus, the changes in sleep spindle duration may reflect maturational alterations in thalamocortical networks across early childhood. Other studies have also shown that age moderates sleep spindle duration. Longitudinal sleep spindle data in infants up to 6 months of age indicate a nonlinear trajectory in spindle duration, such that spindles first increase until 3 months of age and decline thereafter [35]. The average duration of sleep spindles in our sample is shorter (0.85 at 2 Y, 0.91 at 3 Y, and 1.11 at 5 Y) than what was previously reported in infants (2.0 ± 0.3 s at 6 months [35]) and is more similar to adult findings (0.92 ± 0.09 s, ages 20–35 years [6]), although studies have not utilized consistent EEG derivations. In this study, we did not collect data at the age 4 years; however, our findings suggest that the increase in spindle duration during early childhood emerges between ages 3 and 5 years. Further longitudinal data of sleep spindles throughout childhood and adolescence are needed to fully understand the developmental trajectory of spindle duration.

In contrast to other spindle measures, spindle frequency showed early developmental decreases across all EEG regions. Our longitudinal results also indicate an increase in slower frequency sigma activity (e.g., <13.5 Hz) but very few changes in the faster frequencies (i.e., 13.00–13.50 Hz in C4A1 between 2 Y and 5 Y) [15]. These findings differ from those presented in published reports of infants, older children, and adults. A longitudinal study of infants up to 6 months of age found an increase in the percentage of faster frequency sleep spindles with increasing age [35]. Furthermore, cross-sectional data indicate that sleep spindles become faster between late-childhood and adolescence into the adult years [36]. In contrast to our results on developmental changes in sigma power, data from a recent longitudinal analysis of sigma power in 6 to 18 years old revealed a decrease in lower frequency sigma power and an increase in higher frequency sigma power as children grew older [37]. Sigma activity changes between childhood and adolescence are also region-specific and show across night differences [37, 38]. Taken together, our data on maturational changes in both mean spindle frequency and sigma activity extend previous findings in other age groups.

With regard to ISA, we found a developmental increase from 2 Y to 5 Y across all EEG regions (e.g., 56% in C3A2, 93% in C4A1). ISA is an established spindle parameter in adults [11] but has not been investigated longitudinally across early childhood, a period of rapid changes in cognitive abilities such as working memory and mental flexibility (as reviewed in [39]). ISA provides a novel quantification of sleep spindles, and it may be particularly important for future studies of associations between sleep spindles and memory-dependent learning processes. ISA has been linked to overnight memory retention in a word-pair task in adults [11]. Furthermore, spindles may reflect hippocampal-neocortical communication [40], which is an important process for memory consolidation [41, 42]. Because cognitive measures were not assessed in the current study, this proposition remains speculative but represents an area of rich future investigation.

Our longitudinal data suggest that the density of sleep spindles does not change across development. As previously noted, sleep spindle density likely reflects the pace-making activity of the thalamic reticular nucleus [34] and is independent of other spindle characteristics such as ISA, duration, and frequency. The action of different cortical and subcortical mechanisms influences spindle characteristics differently [33, 34]. The maturational changes we observed in sleep spindle duration, frequency, and ISA, but not spindle density, may be a reflection of the nonuniform development of structural brain features [43]. Our results, however, differ from previously published data in children indicating an increase in sleep spindle density in very short bouts (10 min) of NREM sleep during the second half of the nighttime sleep episode [26]. Regarding this discrepancy, we considered the maximum common duration of NREM sleep (688 min), which provided a much longer window for the assessment of spindle density. As the dynamics of sleep spindles change throughout the night and differ between subjects [6, 44], our approach may have resulted in relatively poor resolution of across night differences. Further examination of the all-night temporal aspects of spindle density during childhood is warranted.

### 4.2. Spindle Detection

Our approach extends state-of-the-art methods for spindle identification by showing its utility in young children. The largely subjective and labor-intensive nature of visually scoring sleep spindles is not practical in large datasets and presents unique challenges [2]. For example, sleep spindles are challenging to visually detect in slow wave sleep due to the very high amplitude of slow oscillations in childhood compared to the late adolescent and adult years [8]. In this study, we provide working parameters for automated spindle detection in young children, which makes an important contribution to future research examining sleep spindle development, as well as relationships between spindle characteristics and disease processes across the lifespan [17, 22]. Our findings highlight the strengths (i.e., objective measure, efficiency, and unmasking of spindle features) of using an automated approach and suggest that spindle detection methods are useful tools in basic science and clinical settings.

### 4.3. Strengths, Limitations, and Future Directions

In this study, we assessed healthy children longitudinally at three ages (100% retention), a strong approach for controlling interindividual differences in our measures of interest. We also reduced the likelihood of nuisance variables (e.g., prior sleep history by utilizing a strict sleep schedule, and medications) and employed strict inclusion/exclusion criteria to enroll a healthy homogenous sample. This approach was necessitated by the overarching aims of a larger project examining the early codevelopment and coregulation of sleep homeostasis, circadian rhythms, and emotion processing in early childhood. Thus, our analysis is based upon a relatively small homogenous sample, which limits the generalizability of our findings. Studying a larger community cohort of children, including those with or at risk for disorders such as depression or autism, would extend our findings of sleep spindle maturation and promote the identification of childhood sleep EEG disease biomarkers. Additionally, sleep in early childhood is characterized by a transition from a biphasic to a monophasic sleep-wakefulness schedule. Because our study design restricted napping on the day of overnight sleep recordings, children assessed at younger ages (2 Y and 3 Y) likely accumulated higher sleep pressure across the waking day than at age 5 Y, when they were no longer napping (5 Y) [45]. These dynamic changes could have influenced our measures of sleep spindles and sigma activity. Furthermore, our analytic approach in a relatively small sample may have increased the risk of type 1 errors; however, we utilized a conservative approach to control for multiple comparisons.

Although our age-related adaptation of an automated spindle detection algorithm is novel and further supports using multidimensional approaches to understand sleep spindles and their development across the lifespan, it is not without limitations. Discrepancies between different automated detection algorithms and visual sleep scoring have been reported [2]. Furthermore, low frequency spindle detection (<10 Hz) presents difficulties due to signal attenuation and is difficult to correct (i.e., reducing the frequency cutoff decreases the signal-to-noise ratio, thereby increasing the likelihood of the algorithm detecting waveforms in the alpha and theta range). Thus, the continued fine-tuning of automated spindle detection algorithms remains important to ensure correct identification of electrophysiological phenomena and to allow better between-study comparisons.

Finally, our recordings were restricted to central and occipital EEG derivations. Topographic analysis of sleep spindles in adults supports the existence of “slow” and “fast” sleep spindles over frontal and centroparietal regions [1, 3]. Unfortunately, frontal EEG leads were not used in this study protocol and we therefore avoided separating sleep spindles into slow and fast frequencies. However, few studies have performed regional analysis of sleep spindle frequency in young children and it is possible that children have a dissimilar regional distribution in the frequency of their sleep spindles compared to adults. Further, it is also possible that the cutoff between “slow” and “fast” sleep spindles traditionally applied in adults does not apply in children. It would have been valuable to complete a regional analysis of sleep spindle measures that included frontal EEG derivations.

## 5. Conclusion

In this longitudinal study, we observed developmental changes in several sleep spindle characteristics as well as in slow frequency sigma power across early childhood. Together, our findings indicate that sleep spindle duration, frequency, and ISA may act as sensitive biomarkers for the functional integrity of thalamocortical circuits and for cortical and subcortical brain development. Understanding maturational trajectories of spindles in childhood is important, as it may provide insight into cognitive development and pathology and elucidate key developmental milestones in sleep neurophysiology. Further longitudinal research is needed to understand the functional role of distinct aspects of sleep spindles in structural neurodevelopment and cognitive abilities throughout childhood.

## Figures and Tables

**Table 1:** Descriptive statistics (M, SD) for sleep stage variables at ages 2, 3, and 5 years for the entire nighttime sleep episode. Significant *post hoc* paired *t*-tests (2-tailed) are denoted as ^a^(2 years versus 3 years) and ^b^(2 years versus 5 years).

	2 years		3 years		5 years	
	M	SD	M	SD	M	SD
Time in bed (min)	649.5	12.4	647.4	35.7	635.6	20.2
Total sleep time (min)	602.8	24.4	588.3	28.9	571.9	21.4
Stage 1 (min)	15.8	7.7	26.6	5.9	23.1	12.0
Stage 1 (%)^a^	2.8	1.4	5.0	1.5	4.3	2.1
Stage 2 (min)	272.8	35.8	266.0	36.7	272.9	25.3
Stage 2 (%)	45.3	5.7	45.1	5.0	47.7	3.0
SWS (min)	138.1	26.7	136.0	25.6	134.9	21.0
SWS (%)	22.9	4.3	23.2	4.7	23.7	4.1
REM (min)^b^	174.9	18.6	156.0	21.0	139.7	19.6
REM (%)^b^	29.0	2.6	26.6	3.3	24.4	3.1

**Table 2:** Descriptive statistics (M, SD) of sleep spindle measures at ages 2, 3, and 5 years for each derivation (C3A2, C4A1, O1A2, and O2A1). Significant *post hoc* paired *t*-tests (2-tailed) are denoted as ^a^(2 years versus 3 years), ^b^(2 years versus 5 years), and ^c^(3 years versus 5 years).

	2 years		3 years		5 years		Statistics		
	M	SD	M	SD	M	SD	*F*	*η* ^2^	*p*
Duration (s)									
C3A2^b,c^	0.85	0.13	0.91	0.25	1.11	0.21	9.6	0.26	0.002
C4A1^b,c^	0.79	0.18	0.92	0.29	1.21	0.26	23.5	0.37	<0.001
O1A2^b,c^	0.64	0.05	0.69	0.08	0.94	0.22	12.0	0.51	<0.001
O2A1^b,c^	0.65	0.05	0.67	0.08	0.89	0.17	14.2	0.54	<0.001
Frequency (Hz)									
C3A2^b,c^	13.10	0.20	13.05	0.34	12.77	0.25	13.5	0.25	<0.001
C4A1^b^	13.32	0.37	13.00	0.35	12.72	0.27	11.5	0.39	0.001
O1A2^b^	13.35	0.37	13.10	0.35	12.80	0.26	6.3	0.36	0.010
O2A1^b,c^	13.25	0.32	13.12	0.35	12.77	0.23	7.9	0.34	0.005
ISA (*µ*V)									
C3A2^b,c^	805.5	156.4	905.1	207.8	1253.7	358.2	10.5	0.40	0.002
C4A1^b,c^	704.2	260.9	973.0	512.9	1357.6	454.3	13.5	0.32	<0.001
O1A2^b^	436.4	86.0	626.5	191.5	815.5	228.5	7.4	0.46	0.006
O2A1^b,c^	436.8	67.7	547.0	136.0	778.2	228.1	13.6	0.48	<0.001
Density (#/min)									
C3A2	0.98	0.27	1.08	0.33	0.98	0.21	0.4	0.03	0.67
C4A1	0.93	0.31	1.02	0.40	1.06	0.16	0.7	0.04	0.49
O1A2	0.83	0.47	0.67	0.33	0.55	0.17	1.7	0.11	0.22
O2A1	0.71	0.34	0.54	0.22	0.56	0.19	1.5	0.09	0.25

## References

[1] De Gennaro L., Ferrara M. (2003). Sleep spindles: an overview. *Sleep Medicine Reviews*.

[2] Warby S. C., Wendt S. L., Welinder P. (2014). Sleep-spindle detection: crowdsourcing and evaluating performance of experts, non-experts and automated methods. *Nature Methods*.

[3] Andrillon T., Nir Y., Staba R. J. (2011). Sleep spindles in humans: insights from intracranial EEG and unit recordings. *The Journal of Neuroscience*.

[4] Guazzelli M., Feinberg I., Aminoff M., Fein G., Floyd T. C., Maggini C. (1986). Sleep spindles in normal elderly: comparison with young adult patterns and relation to nocturnal awakening, cognitive function and brain atrophy. *Electroencephalography and Clinical Neurophysiology*.

[5] De Gennaro L., Ferrara M., Bertini M. (2000). Effect of slow-wave sleep deprivation on topographical distribution of spindles. *Behavioural Brain Research*.

[6] Zeitlhofer J., Gruber G., Anderer P., Asenbaum S., Schimicek P., Saletu B. (1997). Topographic distribution of sleep spindles in young healthy subjects. *Journal of Sleep Research*.

[7] Dang-Vu T. T., Schabus M., Desseilles M. (2008). Spontaneous neural activity during human slow wave sleep. *Proceedings of the National Academy of Sciences of the United States of America*.

[8] Ringli M., Huber R. (2011). Developmental aspects of sleep slow waves: linking sleep, brain maturation and behavior. *Progress in Brain Research*.

[9] Chatburn A., Coussens S., Lushington K., Kennedy D., Baumert M., Kohler M. (2013). Sleep spindle activity and cognitive performance in healthy children. *Sleep*.

[10] Lustenberger C., Maric A., Dürr R., Achermann P., Huber R. (2012). Triangular relationship between sleep spindle activity, general cognitive ability and the efficiency of declarative learning. *PLoS ONE*.

[11] Lustenberger C., Wehrle F., Tüshaus L., Achermann P., Huber R. (2015). The multidimensional aspects of sleep spindles and their relationship to word-pair memory consolidation. *Sleep*.

[12] Schabus M., Gruber G., Parapatics S. (2004). Sleep spindles and their significance for declarative memory consolidation. *SLEEP*.

[13] Tamaki M., Matsuoka T., Nittono H., Hori T. (2008). Fast sleep spindle (13–15 Hz) activity correlates with sleep-dependent improvement in visuomotor performance. *Sleep*.

[14] Geiger A., Huber R., Kurth S., Ringli M., Achermann P., Jenni O. G. (2012). Sleep electroencephalography topography and children's intellectual ability. *NeuroReport*.

[15] Doucette M., Kurth S., Chevalier N., Munakata Y., LeBourgeois M. (2015). Topography of slow sigma power during sleep is associated with processing speed in preschool children. *Brain Sciences*.

[16] Shibagaki M., Kiyono S., Watanabe K. (1982). Spindle evolution in normal and mentally retarded children: a review. *Sleep*.

[17] Limoges É., Mottron L., Bolduc C., Berthiaume C., Godbout R. (2005). Atypical sleep architecture and the autism phenotype. *Brain*.

[18] Rauchs G., Schabus M., Parapatics S. (2008). Is there a link between sleep changes and memory in Alzheimer's disease?. *Neuroreport*.

[19] Bove A., Culebras A., Moore J. T., Westlake R. E. (1994). Relationship between sleep spindles and hypersomnia. *Sleep*.

[20] Plante D. T., Goldstein M. R., Landsness E. C. (2013). Topographic and sex-related differences in sleep spindles in major depressive disorder: a high-density EEG investigation. *Journal of Affective Disorders*.

[21] Manoach D. S., Pan J. Q., Purcell S. M., Stickgold R. (2015). Reduced sleep spindles in schizophrenia: a treatable endophenotype that links risk genes to impaired cognition?. *Biological Psychiatry*.

[22] Ferrarelli F. (2015). Sleep in patients with schizophrenia. *Current Sleep Medicine Reports*.

[23] Ferrarelli F., Huber R., Peterson M. J. (2007). Reduced sleep spindle activity in schizophrenia patients. *The American Journal of Psychiatry*.

[24] Göder R., Graf A., Ballhausen F. (2015). Impairment of sleep-related memory consolidation in schizophrenia: relevance of sleep spindles?. *Sleep Medicine*.

[25] Ellingson R. J. (1982). Development of sleep spindle bursts during the first year of life. *Sleep*.

[26] Scholle S., Zwacka G., Scholle H. C. (2007). Sleep spindle evolution from infancy to adolescence. *Clinical Neurophysiology*.

[27] Kurth S., Achermann P., Rusterholz T., LeBourgeois M. (2013). Development of brain EEG connectivity across early childhood: does sleep play a role?. *Brain Sciences*.

[28] Jasper H. H. (1958). The ten-twenty electrode system of the international federation. *Electroencephalography and Clinical Neurophysiology*.

[29] Rechtschaffen A., Kales A. (1968). *A manual of Standardized Terminology, Techniques and Scoring System for Sleep Stages of Human Subjects*.

[30] Huupponen E., Värri A., Himanen S.-L., Hasan J., Lehtokangas M., Saarinen J. (2000). Optimization of sigma amplitude threshold in sleep spindle detection. *Journal of Sleep Research*.

[31] Nir Y., Staba R. J., Andrillon T. (2011). Regional slow waves and spindles in human sleep. *Neuron*.

[32] Benjamini Y., Hochberg Y. (1995). Controlling the false discovery rate: a practical and powerful approach to multiple testing. *Journal of the Royal Statistical Society*.

[33] Bonjean M., Baker T., Lemieux M., Timofeev I., Sejnowski T., Bazhenov M. (2011). Corticothalamic feedback controls sleep spindle duration in vivo. *The Journal of Neuroscience*.

[34] Fuentealba P., Steriade M. (2005). The reticular nucleus revisited: intrinsic and network properties of a thalamic pacemaker. *Progress in Neurobiology*.

[35] Louis J., Zhang J. X., Revol M., Debilly G., Challamel M. J. (1992). Ontogenesis of nocturnal organization of sleep spindles: a longitudinal study during the first 6 months of life. *Electroencephalography and Clinical Neurophysiology*.

[36] Nicolas A., Petit D., Rompré S., Montplaisir J. (2001). Sleep spindle characteristics in healthy subjects of different age groups. *Clinical Neurophysiology*.

[37] Campbell I. G., Feinberg I. (2016). Maturational patterns of sigma frequency power across childhood and adolescence: a longitudinal study. *SLEEP*.

[38] Kurth S., Ringli M., Geiger A., LeBourgeois M., Jenni O. G., Huber R. (2010). Mapping of cortical activity in the first two decades of life: a high-density sleep electroencephalogram study. *Journal of Neuroscience*.

[39] Buss A. T., Spencer J. P. (2014). The emergent executive: a dynamic field theory of the development of executive function. *Monographs of the Society for Research in Child Development*.

[40] Gais S., Mölle M., Helms K., Born J. (2002). Learning-dependent increases in sleep spindle density. *The Journal of Neuroscience*.

[41] Wang S.-H., Morris R. G. M. (2010). Hippocampal-neocortical interactions in memory formation, consolidation, and reconsolidation. *Annual Review of Psychology*.

[42] Saletin J. M., Walker M. P. (2012). Nocturnal mnemonics: sleep and hippocampal memory processing. *Frontiers in Neurology*.

[43] Sowell E. R., Trauner D. A., Gamst A., Jernigan T. L. (2002). Development of cortical and subcortical brain structures in childhood and adolescence: a structural MRI study. *Developmental Medicine and Child Neurology*.

[44] Himanen S.-L., Virkkala J., Huhtala H., Hasan J. (2002). Spindle frequencies in sleep EEG show U-shape within first four NREM sleep episodes. *Journal of Sleep Research*.

[45] Jenni O. G., LeBourgeois M. K. (2006). Understanding sleep-wake behavior and sleep disorders in children: the value of a model. *Current Opinion in Psychiatry*.

